# Perception and manifestation of collaborative competencies among undergraduate health students*

**DOI:** 10.1590/1518-8345.3227.3240

**Published:** 2020-02-03

**Authors:** Ana Wládia Silva de Lima, Fábia Alexandra Pottes Alves, Francisca Márcia Pereira Linhares, Marcelo Viana da Costa, Maria Wanderleya de Louvor Coriolano-Marinus, Luciane Soares de Lima

**Affiliations:** 1 Universidade Federal de Pernambuco, Centro Acadêmico de Vitória, Vitória de Santo Antão, PE, Brazil.; 2 Universidade Federal de Pernambuco, Centro de Ciências da Saúde, Recife, PE, Brazil.; 3 Universidade Federal do Rio Grande do Norte, Escola Multicampi de Ciências Médicas, Caicó, RN, Brazil.

**Keywords:** Interprofessional Relations, Professional Training, Professional Competence, Higher Education, Primary Health Care, Integrality in Health, Relações Interprofissionais, Capacitação Profissional, Competência Profissional, Educação Superior, Atenção Primária em Saúde, Integralidade em Saúde, Relaciones Interprofesionales, Capacitación Profesional, Competencia Profesional, Educación Superior, Atención Primaria de Salud, Integralidad en Salud

## Abstract

**Objective:**

to analyze the perception and manifestation of collaborative teamwork competencies among undergraduate health students who experienced the curricular internship’s integration module from the perspective of interprofessional education.

**Method:**

qualitative study, developed with the intervention research strategy. Twenty-eight students from five undergraduate health courses participated. Data were collected in three focus group interviews conducted with the undergraduate students at the end of each semester. For data analysis, the technique of intervention research and dialectical hermeneutics adopted was based on the theoretical framework of interprofessional education in health.

**Results:**

uniprofessional culture, the experience of integration of different fields of knowledge and collaborative competencies were manifested by the students in their reports and in the actions developed by the multidisciplinary team with individuals and families, during the experience of the curricular internship’s integration module.

**Conclusion:**

the experience of integration of the curricular internship from the perspective of interprofessionality favored the perception and manifestation of collaborative competencies that are necessary for teamwork among the students.

## Abstract (pt)

**Objetivo:**

analisar a percepção e manifestação de competências colaborativas para o trabalho em equipe entre discentes de graduação em saúde que vivenciaram o módulo integrador do estágio curricular na perspectiva da educação interprofissional.

**Método:**

estudo qualitativo, desenvolvido com a estratégia da pesquisa-intervenção. Participaram 28 discentes de cinco cursos de graduação em saúde. Os dados foram apreendidos em três grupos focais realizados com os discentes da graduação ao termino de cada semestre letivo. Para a análise dos dados adotou-se a técnica da pesquisa-intervenção e da hermenêutica dialética à luz do referencial teórico da formação em saúde, da educação interprofissional.

**Resultados:**

a cultura uniprofissional, a experiência da integração das diferentes formações e as competências colaborativas foram perceptíveis e manifestadas pelos discentes nos relatos e nas ações em equipe multiprofissional, desenvolvidas com os indivíduos e as famílias, durante a experiência do módulo integrador do estágio curricular.

**Conclusão:**

a experiência de integração do estágio curricular na perspectiva da interprofissionalidade favoreceu nos discentes a percepção e manifestação de competências colaborativas necessárias para o trabalho em equipe.

## Abstract (es)

**Objetivo:**

analizar la percepción y manifestación de competencias colaborativas en el trabajo en equipo entre estudiantes de grado en salud, que tuvieron experiencia en el módulo integrador de las prácticas curriculares desde la perspectiva de la educación interprofesional.

**Método:**

estudio cualitativo, desarrollado con la estrategia de investigación-intervención. Participaron 28 estudiantes de cinco cursos de grado en salud. Se recolectaron los datos de tres grupos focales que han sido realizados con estudiantes de grado al final de cada semestre académico. Para el análisis de datos, se utilizó la técnica de investigación-intervención y de la hermenéutica dialéctica a la luz del marco teórico de la formación en salud y de la educación interprofesional.

**Resultados:**

la cultura uniprofesional, la experiencia de la integración de las diferentes formaciones y las competencias colaborativas fueron notables y manifestadas por los estudiantes en sus relatos y acciones en equipo multiprofesional, desarrolladas con individuos y familias, durante la experiencia del módulo integrador de las prácticas curriculares.

**Conclusión:**

la experiencia de integración de las prácticas curriculares en la perspectiva de la interprofesionalidad les favoreció a los estudiantes con la percepción y manifestación de competencias de colaboración necesarias para el trabajo en equipo.

## Introduction

The health education model in Brazil happens in a hegemonic, uniprofessional and disciplinary way, based on the regulations of professions related to market restraints, and focused on the pathophysiological conception of life^(^ 1 ^-^ 3 ^)^. This model is reproduced in the work process of these professionals, with isolated practices that do not favor teamwork, subjectifying individuals/patients and creating a hierarchy of actions in health, in growing disagreement with the complex needs of the increasingly interconnected contemporary world^(^ 3 ^-^ 6 ^)^.

In 1998, the World Health Organization, the Pan American Health Organization, universities and researchers around the world listed several macro, meso, and micro-policy actions needed to readjust the model of education in health^(^ 6 ^-^ 9 ^)^.

Among the suggestions of changes at the meso-political level involving curricular adaptations and more active methodological strategies, Interprofessional Education (IPE) has been used in the readjustment of the model of education and professional practice, meeting the health needs of the population by developing the collaborative teamwork competencies of health professionals, enhancing the effectiveness and quality of the care provided^(^ 2 ^-^ 9 ^)^.

The competency-based training model emerges at the beginning of the twentieth century due to a demand from the labor market^(^ 10 ^-^ 11 ^)^, in an attempt to qualify professionals to solve unexpected problems, promoting learning competencies and mobilizing knowledge, attitudes and expanded and plastic skills that are able to address the numerous and different needs of society^(^ 11 ^)^. Moreover, the development of competencies is associated with meaningful learning, whereby individuals build emotional memories experienced in their learning process^(^ 10 ^)^, which requires revising the teaching-learning process focused on the student and on practical applications of knowledge^(^ 3 ^,^ 10 ^-^ 13 ^)^.

IPE is conceptualized as integrated and interactive learning between two or more health professions, allowing a greater understanding of the specific roles of each professional and enhancing the development of collaborative teamwork competencies^(^ 14 ^-^ 15 ^)^. Thus, it is understood that experimentation and interactive living may promote the development of collaborative competencies.

Systematic reviews involving studies in the last decade have analyzed the profile of students subjected to IPE experiences, and describe changes in the development of collaborative teamwork competencies, such as: development of values and ethics for the promotion of humane care practices, better communication between team members, and identification and recognition of professional roles, enhancing the level of respect between professional categories and favoring the complementarity, quality and safety of care^(^ 16 ^-^ 17 ^)^. However, collaborative competencies do not develop immediately, they require continuous practice and interprofessional collaboration. Competencies such as ethics/values and professional roles are less developed than communication and teamwork^(^ 16 ^-^ 17 ^)^.

This evidence substantiated research groups and associations from different medical fields, which have been identifying and consolidating collaborative skills, knowledge and attitudes, building panels and matrices of collaborative competencies based on global IPE experiences, such as the Canadian Interprofessional Health Collaborative (CICH) and the North American Interprofessional Education Collaborative (IPEC). These panels have been strongly recommended as a way to promote the insertion of these competencies in the training curricula, so the students’ profile may be evaluated at a later time for the accreditation of interprofessional learning experiences^(^ 18 ^-^ 19 ^)^.

**Figure 1:**
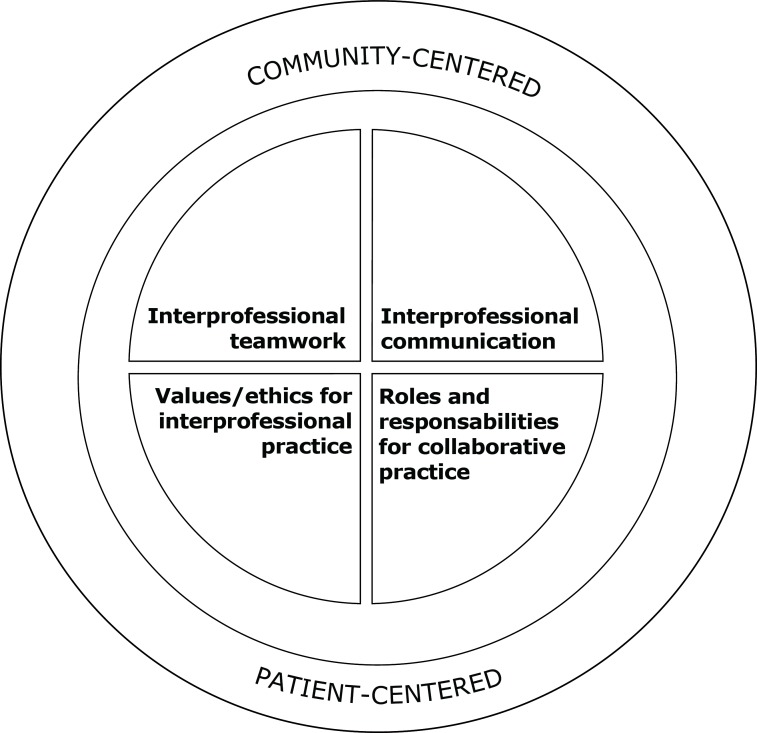
Core competencies for interprofessional collaborative practice IPEC^1^

In 2016, IPEC published an update of its competency panel, which is organized into two core principles - community-centered care and patient-centered care. These involve four key domains/competencies: values/ethics for interprofessional practice; roles and responsibility for collaborative practice; inter-professional communication, and; inter-professional teamwork (Figure 1). The four domains/competencies include 42 sub-competencies that comprise the theoretical axis of each domain^(^ 19 ^)^.

However, the effectiveness of IPE in the development of these competencies still needs more robust evidence. The literature recommends the performance of studies with higher quality and methodological rigor in relation to the measurement of the development of competencies, using mixed and complementary methodological approaches^(^ 15 ^-^ 22 ^)^.

Assessing the effectiveness of learning is among the needs of organizations that invest in Training, Development and Education (TD&E). The literature in this area indicates three important categories as predictors of the results: the (motivational, cognitive, affective, professional) characteristics of the trainees; of the instructional design and its delivery; and of the organizational context (support, climate, culture, before, during and after training)^(^ 23 ^)^.

Considering this context, this study aimed to analyze the perception and manifestation of collaborative teamwork competencies among students from different health backgrounds whose curricular internship featured collaborative experiences, using a qualitative methodological proposal that is consistent with the proposal of interprofessional education.

## Method

This is a qualitative study using intervention research (IR) as method of choice. In this methodological strategy, intervention and analysis are carried out concurrently: at the same time theory and practice are reflected upon and problematized, the actors are mobilized in a movement of implication, leading them to collectively identify the needs for personal and institutional changes/transformations, a process that is aligned with the proposal of reflection of IPE regarding the articulation and implication of different macro, meso and micro institutional actors for its implementation and effectiveness in the readjustment of the training model and of the practice of health professionals^(^ 9 ^-^ 14 ^,^ 24 ^-^ 25 ^)^.

**Figure 2:**
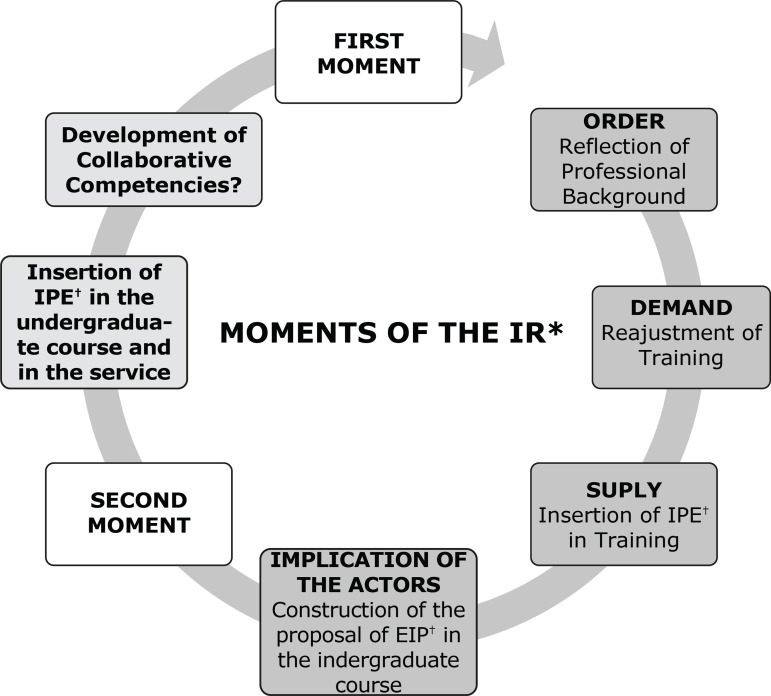
Moments of the Intervention Research *Intervention Research; †Interprofessional Education

IR uses specific concepts to understand the social reality studied, deconstructing the practices and discourses established, in a process of mobilization and transformation of reality^(^ 26 ^-^ 27 ^)^. This IR was developed in two sequential moments, as described in Figure 2.

The IR’s development in two moments aimed to mobilize the different actors (professors, health professionals, students), encouraging them to reflect upon and discuss the model of professional training in health to build a collective instructional proposal, integrating teaching and service, so that its implementation in the course’s curricula and in the practice of the services would not happen in a vertical and decontextualized manner^(^ 3 ^,^ 9 ^)^.

The first moment corresponded to the IR’s ideation, with a reflection on the scenario of practice and professional education, triggering the order, followed by the demand, the need to readjust the model of training of health professionals, with interprofessional education as strategic device. To meet this demand, the proposal of insertion of IPE in health courses was supplied. This whole process required the mobilization of the different institutional actors in meetings and workshops, having as final product the proposal of an integration module to be inserted in the curricular internship of the undergraduate courses in health. The second moment of the IR corresponded to the direct experimentation of the proposal by the students. This article analyzed this second moment of the IR.

Twenty-eight undergraduate students participated, four from the course in physical education (bachelor’s degree), and six each from the courses in nursing, nutrition, public health (bachelor’s degree) and biological sciences (licentiate). These students were selected in an open competition promoted by PET-Saúde Gradua SUS, under which this research was conducted, as fellowship holders^(^ 12 ^)^ and volunteers^(^ 16 ^)^. As inclusion criterion, the student should have been enrolled in the semester of the compulsory internship of his/her course, which should have taken place in the Family Health Strategy (FHS) of the Family Health Support Center (NASF), for the bachelor’s degrees, and in the School of Basic Education, for the licentiate. This IR was developed in the second half of 2016, the first half of 2017 and the first half of 2018.

Each semester, the students were distributed in two multidisciplinary teams to work in two different practical scenarios, located in a health territory linked to the institution. This territory was selected considering the criteria of teaching-service integration and adherence to the Program of Education in Health through Work - PET-Saúde since 2010.

The curricular internship’s integration module corresponded to 80 hours per semester, being held on a fixed day of the week in both training scenarios. The activities of the multiprofessional student teams, developed both at the FHS/NASF and at the school, had as instructional proposal following IPEC’s core principles (2016)^(^ 19 ^)^, i.e., providing community-centered care and patient-centered care. During the work process, the students needed to always work in a multiprofessional team, made up of at least two students from different backgrounds. The practical activity in the FHS scenario began with the recognition of this territory, of a micro area and, as per indication of the FHS’s team, of three families with complex needs.

The student team, in home visits, began the process of bonding with the families and their components and recognizing the area and micro-area. The collaborative work process was then activated for the construction of the diagnosis and of the integrated care plan to be developed with the family or a specific member of it. This plan was reviewed every two weeks by the student team together with the family/individual. This whole process was guided by preceptors (health professionals of the FHS and NASF) and tutors (professors of the institution) during the direct and indirect supervisions of the curricular internship.

Data were produced in three focus group interviews conducted with the multidisciplinary student teams at the end of each semester. The interviews lasted an average of two hours and were mediated by the researcher with the help of a list of questions that contemplated the structure of the integration module (critical evaluation of the integrated internship), the process (how the team activities were developed), and the results (the understanding of the professional roles and of the multidisciplinary actions developed with the family). The mediator acted by making connections between reports, favoring the free dialogue between the students. The focus group interviews had an external collaborator, who acted in the control of the image and voice recording equipment and as rapporteur, registering the most relevant topics and aspects of the discussion and of nonverbal communication. At the end of each interview, the topics and records were read by the rapporteur and validated by the participants. The images and reports were recorded using a Sony Splashproof exmor R camera.

The perception and manifestation of the collaborative competencies were obtained from the students’ reports about their experience with the curricular internship’s integration module. The reports were organized by semester, and subsequently sorted according to the core principles and domains/competencies of IPEC (2016)^(^ 19 ^)^ that were used as thematic axes in this research.

For the analysis of the reports, the techniques of intervention research and comprehensive reading of dialectical hermeneutics^(^ 24 ^-^ 28 ^)^ were adopted, with the aim of understanding the analyzers that emerged in actions and/or discourses throughout the process, from the perspective of the theoretical-conceptual frameworks of education in health and interprofessional education.

The study was approved by the Research Ethics Committee of the institution studied, CAEE No. 55947616.3.0000.5208, under opinion No. 2.669.748. To preserve the participants’ confidentiality, acronyms were used to designate them according to undergraduate course: Nursing (NS), Collective Health (CHS), Physical Education (PES), Nutrition (NutS) and Biological Sciences (BSS), followed by numbers indicating the sequence of insertion in the study.

## Results

The socio-demographic profile of the students revealed that 93% lived in municipalities of Pernambuco. The mean age was 22 years old; five were male, and 23, female. Most (22) students were in the last semester of the undergraduate course, and 16 reported previous experience with programs developed by the student movement and extension programs of the community and service, such as (VERSUS) - Experiences and Internships in the Reality of the Brazilian Unified Health System and (PET-Health) - Program of Education in Health through Work.

The students’ reports are described below, considering the organizational process and the development of the curricular internship’s integration module, followed by the classification of these reports according to the principles and domains/competencies of IPEC, recognized for collaborative interprofessional practice and understood, in this research, as thematic axes.

At the beginning of the curricular internship’s integration module, there was some discomfort with the formation of multiprofessional teams composed of one student from each area, especially when the lack of academic experience in studying/collaborating with professionals from other areas was perceived. I thought it was awful, awful! When this was said I thought, what now, I became tense and scared. We had not yet had the opportunity to work with others from different courses, I had not had it. I considered giving up (BSS3).

Throughout the experience, these discomforts diminished, reducing the strangeness with the experimentation of integration. We used to be more distant from each other, you know? Like, each doing his or her own thing. It’s a shame it’s ending, I think it worked well, we managed to achieve all goals (NS2). The team’s resourcefulness is a lot more noticeable today, which is normal, right? (BSS1). Now I get it, after going through the whole process I get it, I can work better and I learned from the experience, it’s easier for me, I can now see that it’s possible to work like this with different groups, because it’s so much easier to work with people who think the same way we do, right? (BSS3).

The moment of the curricular internship in primary care and basic education was viewed as opportune, allowing the effective integration between the different areas. Who would have known this group would end up working so well, because at the university we don’t work like that, we don’t even see each other. Nursing classes are in the afternoon, nutrition and physical education are in the morning, and at night we can’t have a combination of these courses, right? Working together, practicing as a team, because the internship that is exclusive to your course is one thing, whereas this is totally different, and how much I’ve already learned here is unexplainable (CHS6). What was most significant in the internship experience was the cooperation, the exchange of knowledge between courses, and being able to realize that, in a way, one complements the other. We are there with different backgrounds, but a single purpose (NutS4).

It was identified, however, that the workload established for the internship’s integration module of 80 hours in the semester should be increased, allowing greater exposure to the experience. At first we had a skeptical vision of the proposal, but halfway through the internship was when I really understood what it would be like (BSS3). For me, I felt out of focus, I didn’t understand my role, but now I can see each one’s role here (CHS1).

The manifestation and perception of competencies were evidenced in the following reports, described sequentially in the thematic axes established for the research.

Principles: Community-centered care and patient-centered care - core principles that broaden the insight into the needs of the community and of the patient/family. With the visits we were able to see the complexity of the family, like, we generalized it, saw the social and other needs (CHS6). We want to focus only on what is within our comfort zone, but being exposed to different knowledge makes us see that we can reach the family in a much broader manner (PES4). I think that we can both serve the families in a more holistic way and make them feel more cared for in this sense (NS6). We realize that health encompasses everything, including social, psychological and physiological needs, the latter being our main focus in the academia. We are conditioned to only think about the pathology itself, and when we experience the daily reality of the community, we see that it’s not just the health-disease process, there’s a lot going on backstage, far beyond what we think and see in college (CHS6).

Competency 1 - Values/ethics for interprofessional practice: work with people from other professions to maintain a climate of mutual respect, share values, realize the real needs of individuals. There are so many different professional perspectives, so what I do not realize, she is able to catch on, and when we come to the Unit after the visit, to make the care plan, then we begin to articulate: I thought of doing so-and-so, then someone else says, but this is what I observed, so when we come together it really gets much broader (NS6). It’s also no use talking to the family if they have no interest in putting it into practice, there needs to be interest on their part (NutS6).

Competency 2 - Professional roles and responsibilities for collaborative practice: recognize one’s own role and that of other professions to properly assess and meet the patients’ health care needs. I, as a nurse, want to do what is best for the patient on that visit, but there’s some things that even if I do my best, if there isn’t another professional from some other area, I can try and do some of what he does, but I can’t do it like he does it, so once again, the importance of teamwork shows (NS5). I had never experienced working together with another professional, so I could see her ability added to mine, for the patient’s well-being (PES4). We have some misconceptions, that nutrition is this, physical education is just that. We play around a lot in college, that physical education should stay on the court, that it’s just playing ball (laughs), and when we’re together like this, we see the importance of each of us and that it goes beyond what we had imagined. This third family was a great example, since I thought, oh gee, a bedridden lady, what physical activity would she even be able to do, then on the visit, (PES) nailed it, a bedridden lady is going to exercise more than I do (laughs). I had no such vision, I couldn’t imagine everything he put into practice (NS6).

Competency 3 - Interprofessional communication: communicate with patients, families, communities and health professionals, in an agile and responsible manner, and resolve interpersonal conflicts within the team, for the treatment of the disease and prevention, promotion and maintenance of health. Another thing I realized is that we need to talk, because if you have a problem you have to try and solve it, and you can only do that by talking and looking for solutions, right? It’s no use having a problem and not talking about it. The situation we experienced was an example of that, right? We had to stop and discuss what was hindering the progress of the team’s actions (CHS2).

The language used with the patient is an extremely important communicational competency for integrating the individual and family into their own care. In the following report, it may be noted that the language used needed to be adapted for the patients’ comprehension. So, you need to think of how to speak, use a language that they can understand. You get the hang of it in practice, when in contact with the patient’s reality. I used to say, do it as if on a crucifix, remember that? Open your arms like Christ on the cross. I always make an association, try to associate it with something (PES3).

Competency 4 - Interprofessional teamwork: apply the relationship of value building and team dynamics, effectively executed between different professions, to plan, offer, and evaluate patient/community-centered care plans involving different competencies. After visiting the family, we get together and build the plan, each one gives their general opinion based on the knowledge they have, and we keep on developing this plan to then implement it in the family (NS6). We finish the first visit and need a second, sometimes third one to plan what to do. While at home, we think of other things, of how to improve. We’d communicate on other days also, not only on the day of the internship, on whatsApp, to exchange ideas (NutS6). At the time of the diagnosis, there are things that she can see that I hadn’t noticed. The shared construction, this multidisciplinarity of working together, the importance of construction itself, its impact could not be any different, it’s positive because the intervention becomes much more comprehensive (NS3).

## Discussion

The uniprofessional model is naturalized in the training and work process of health professionals. The initial apprehension experienced by the students is characteristic of this model of disciplinary training, in which specific competencies are highly valued^(^ 1 ^,^ 3 ^,^ 6 ^,^ 12 ^-^ 13 ^)^, leading some students to show discomfort in working with professionals from other areas. This difficulty is understandable, considering the uniprofessional background of all involved (students, professors, professionals of the services), wherein opportunities for action and integrated learning with professionals from other areas are sporadic, offered almost exclusively in extracurricular activities^(^ 29 ^-^ 30 ^)^. To improve their understanding of the model, it was noted that the integration module’s workload should be expanded.

The literature indicates the importance of understanding the characteristics of learners, since their knowledge, implications and motivations can influence the results^(^ 23 ^)^. The profile of the students, mostly young women, with previous experience with multiprofessional projects and involved in the political student movement, reveals their motivations and implications in relation to proposals to be implemented, which may have been a positive factor of the results.

The ideal time for interprofessional experience is not a consensus. Some authors recommend it takes place at the beginning of the course, in order to reduce prejudiced stereotypes^(^ 3 ^,^ 12 ^,^ 14 ^-^ 15 ^)^; other authors recommend it takes place at the end, given that by then the specific competencies of each course have already been consolidated, enabling greater interaction between the students from different areas, who better understand their own professional role and that of others^(^ 31 ^)^. However, it is understood that the experience of interprofessional education should be offered according to the possibilities of effective integration between students, considering the characteristics of each institution^(^ 3 ^,^ 12 ^-^ 15 ^)^. In this research, the moment of the curricular internship was perceived as viable and effective for the integration between the different courses, due to the HEI’s infrastructure.

Collaborative teamwork competencies gradually materialized in the mutual interactions between students, patients/family and the community, from the perspective of interprofessional education. Attention is drawn to the interrelationship between competencies, not considering individual manifestations, but rather their coexistence in the analyzed reports.

According to the literature, centering care on the patient and on the community, with ethical apprehension of the concrete needs of patients/family, allows students to develop a better perception of the comprehensiveness of care, leading them to recognize the need for the complementarity of knowledge^(^ 2 ^,^ 5 ^,^ 12 ^,^ 16 ^-^ 17 ^,^ 20 ^)^.

The reports show that the students acknowledged the individual and collective needs of individuals and families, based on the reality observed. The implementation of the care plan with the integration of knowledge and practices of each area around a common goal allowed them to realize the importance of other professions and the complementarity between areas, as they learned from and about the others and recognized the different professional roles.

Similar results integrating different areas in patient-centered community practice were obtained by IPE programs of undergraduate health courses in various HEIs around the world^(^ 32 ^-^ 34 ^)^. A recent systematic review highlighted that IPE programs in which team action is based on practice favored the effective development of IPE and, consequently, collaborative competencies^(^ 16 ^)^. The characteristics of the training program are indicated as one of the predictors of positive or negative results in several TD&E programs^(^ 23 ^)^.

Developing altruistic attitudes is related to the sense of implication, of putting oneself in another’s place^(^ 35 ^)^. The proposal of centering care on the family’s reality stimulated the shift of focus from specialized care to extended care, as predicted by the researchers^(^ 18 ^-^ 19 ^)^. In the reports presented, the students perceive the patients’ needs beyond their specific competency, developing an understanding of the social role that health professionals should assume, as highlighted by the CICH and IPEC panels^(^ 18 ^-^ 19 ^)^.

Values and ethics as competency for collaborative interprofessional practice also becomes evident when focusing care according to IPEC’s core principles^(^ 19 ^)^. Teaching based on team practice awakens the future professional’s perception of his/her ethical social role^(^ 16 ^-^ 17 ^)^, similarly to what was evidenced in the reports of this research. The experience of acting in a multidisciplinary team in primary care through home visits allowed the students to share their perspectives and recognize the social context in which the families live, favoring the construction of an integrated care plan that is ethically focused on the needs found and on the family’s capacity of involvement. Similar studies where the student teams’ work is focused on the patient show that there is greater perception on the part of the students, beyond their specific competencies^(^ 12 ^,^ 32 ^-^ 34 ^)^.

The recognition of the roles and responsibilities involved in collaborative practice was evidenced in several reports of the students of this research. The development of respect for the other professionals in training through the experience of learning together favors the elimination of stereotypes^(^ 16 ^,^ 32 ^-^ 34 ^,^ 36 ^-^ 38 ^)^. In the students’ reports, the perception of their own professional role and that of the others was present, corroborating the proposal of IPE^(^ 14 ^-^ 15 ^)^ and the studies that have shown the development of this competency in the interrelationships between professionals from different areas^(^ 12 ^-^ 19 ^,^ 29 ^-^ 34 ^,^ 36 ^-^ 38 ^)^.

Not transcending the barrier of specific competencies was evidenced in the reports of the student who demonstrates understanding that specificity is exclusive, however, general and generic orientations can be shared and developed by all. Similar data corroborate this finding, with expansion and optimization of the comprehensive care of patients and their families by the interprofessional team^(^ 18 ^-^ 19 ^,^ 36 ^-^ 38 ^)^.

Knowing how to dialogue, expressing oneself in a way that does not create discomfort or misunderstandings, is fundamental to minimize conflicts in the workplace. Communicative competency is one of the most important for collaborative teamwork, requiring common sense, experience, and the parallel development of attitudes such as respect, trust and unity within the team^(^ 39 ^-^ 42 ^)^.

Interprofessional communication is essential for collaboration, and is present in professional-professional and professional-patient/family interactions, being necessary to develop effective and understandable exchanges in different fields of action, and also to resolve conflicts, promoting harmony in the team^(^ 40 ^-^ 42 ^)^. Knowing how to speak, how to listen and how to respect differences was an attitude experienced by the students during the curricular internship’s integration module.

The situation of conflict highlighted in one student’s report was related to interpersonal relationships and installed a climate of tension within the team, requiring the mobilization of communicative attitudes and skills for its resolution. The public health student took the initiative in the conflict’s management, possibly due to his professional profile. Dealing with conflicts and identifying a better solution is a sub-competency of the interprofessional communication domain, highlighted in both the IPEC panel (2016) and the CICH panel for the resolution of conflicts within teams^(^ 18 ^-^ 19 ^)^.

Still within the domain of communication, the interaction with patients, learning to communicate with them and using a language that is compatible with their cultural reality, was perceived by the students as necessary to establish effective and understandable exchanges with patients and their families, corroborating studies that highlight this importance^(^ 41 ^-^ 42 ^)^.

The interprofessional teamwork competency - which relates to team dynamics and functioning, aggregating the other competencies and interrelating them - is developed according to the literature, during training and in the continuous practice of interprofessional collaboration, in a progressive process of apprehension and sharing of common goals^(^ 3 ^,^ 14 ^-^ 15 ^,^ 18 ^-^ 19 ^,^ 43 ^-^ 44 ^)^.

The teamwork dynamics was experienced and built in the visits to the patients, and it was noted, in the students’ reports, that learning to work together was a process that was mutually developed by all the teams in collaborative actions, in the different scenarios and families. These experiences promoted the students’ learning and integration, with IPE having also been observed outside the intervention scenarios, allowing the continuous sharing of ideas through social networks, similarly to other studies^(^ 3 ^,^ 15 ^)^.

The students’ collaborative action was developed by combining different knowledge and practices for the construction of the integrated care plan, with the sharing of common and specific competencies and manifestation of collaborative competencies in a procedural and continuous way. It was noted that the team acquired better resourcefulness over the period of the experience, and also that the nursing student frequently assumes a leadership role, which is recognized and allowed by his/her colleagues.

Factors related to the local context limited the development of the curricular internship’s integration module. The high demand for the FHS/NASF by the public and private HEIs of the municipality hindered the common start of the internships, with differences in the time of entry of the students in the field of practice. Also, the local political influence and the precarious contractual bond of the health network’s professionals caused frequent changes of preceptors, requiring new implication and awareness-raising measures.

## Conclusion

The study identified perceptions and manifestations of collaborative teamwork competencies in the reports of students from different health backgrounds about the experience of interprofessional education during the curricular internship’s integration module.

This research, using IR as significant methodological strategy for the implication and involvement of different social actors, has also corroborated the viability of the institutional proposal of IPE in pedagogical projects, for insertion of the integration module in the curricular matrix of each undergraduate health course in the reality studied.

## Footnotes

* Interprofessional Education Collaborative^(^ 19 ^)^ - This document may be reproduced, distributed, publicly displayed and modified provided that attribution is clearly stated on any resulting work and it is used for non-commercial, scientific or educational — including professional development — purposes. If the work has been modified in any way all logos must be removed
